# Effects of carbamazepine and lamotrigine on functional magnetic resonance imaging cognitive networks

**DOI:** 10.1111/epi.14448

**Published:** 2018-06-13

**Authors:** Fenglai Xiao, Lorenzo Caciagli, Britta Wandschneider, Josemir W. Sander, Meneka Sidhu, Gavin Winston, Jane Burdett, Karin Trimmel, Andrea Hill, Christian Vollmar, Sjoerd B. Vos, Sebastien Ourselin, Pamela J. Thompson, Dong Zhou, John S. Duncan, Matthias J. Koepp

**Affiliations:** ^1^ Department of Clinical and Experimental Epilepsy University College London Institute of Neurology London UK; ^2^ Department of Neurology West China Hospital of Sichuan University Chengdu Sichuan China; ^3^ Magnetic Resonance Imaging Unit Epilepsy Society Gerrards Cross UK; ^4^ Department of Neurology Medical University of Vienna Vienna Austria; ^5^ Department of Neurology Epilepsy Center University of Munich Munich Germany; ^6^ Wellcome/Engineering and Physical Sciences Research Council Centre for Interventional and Surgical Sciences University College London London UK; ^7^ Translational Imaging Group University College London London UK

**Keywords:** antiepileptic drugs, carbamazepine, cognition, functional MRI, lamotrigine

## Abstract

**Objective:**

To investigate the effects of sodium channel–blocking antiepileptic drugs (AEDs) on functional magnetic resonance imaging (fMRI) language network activations in patients with focal epilepsy.

**Methods:**

In a retrospective study, we identified patients who were treated at the time of language fMRI scanning with either carbamazepine (CBZ; n = 42) or lamotrigine (LTG; n = 42), but not another sodium channel–blocking AED. We propensity‐matched 42 patients taking levetiracetam (LEV) as “patient‐controls” and included further 42 age‐ and gender‐matched healthy controls. After controlling for age, age at onset of epilepsy, gender, and antiepileptic comedications, we compared verbal fluency fMRI activations between groups and out‐of‐scanner psychometric measures of verbal fluency.

**Results:**

Patients on CBZ performed less well on a verbal fluency tests than those taking LTG or LEV. Compared to either LEV‐treated patients or controls, patients taking CBZ showed decreased activations in left inferior frontal gyrus and patients on LTG showed abnormal deactivations in frontal and parietal default mode areas. All patient groups showed fewer activations in the putamen bilaterally compared to controls. In a post hoc analysis, out‐of‐scanner fluency scores correlated positively with left putamen activation.

**Significance:**

Our study provides evidence of AED effects on the functional neuroanatomy of language, which might explain subtle language deficits in patients taking otherwise well‐tolerated sodium channel–blocking agents. Patients on CBZ showed dysfunctional frontal activation and more pronounced impairment of performance than patients taking LTG, which was associated only with failure to deactivate task‐negative networks. As previously shown for working memory, LEV treatment did not affect functional language networks.

**Key Points**

- A verbal fluency functional MRI approach was employed to investigate the effects of carbamazepine and lamotrigine on cognition
- Those on carbamazepine showed less activation in left inferior frontal gyrus compared to those taking levetiracetam or healthy controls
- Those on lamotrigine had more failed task‐related deactivations compared with those on levetiracetam or healthy controls
- Patients on carbamazepine showed dysfunctional frontal activation and more pronounced impairment of performance than those on lamotrigine

## 1 INTRODUCTION

Lamotrigine (LTG) and carbamazepine (CBZ) are both effective antiepileptic drugs (AEDs) and well‐tolerated in epilepsy patients.^1^ Cognitive deficits commonly associated with AED therapy are rarely observed in patients receiving LTG therapy, whereas CBZ produces modest negative untoward effects.^1^, ^2^ Direct comparison of the 2 AEDs revealed significantly better cognitive performance on LTG than CBZ.^1^ It remains unclear how these 2 widely used antiepileptic drugs specifically affect cognition in patients with epilepsy.

In cognitive functional magnetic resonance imaging (fMRI), consistent and reproducible patterns of activation and deactivation are elicited by goal‐directed tasks, including language and memory. The default mode network (DMN) represents a set of brain regions that is commonly deactivated during goal‐directed tasks and has been traditionally associated with mind‐wandering and envisioning the future.^3^, ^4^ The verbal fluency fMRI task usually elicits the activation of frontal lobe areas, particularly the dominant inferior frontal gyrus (IFG), middle frontal gyrus (MFG), anterior cingulate, and precentral cortices, as well as the insular, superior temporal, and parietal cortices and the cerebellum.^5^, ^6^, ^7^ Its relatively low demand makes it an important tool for the assessment and lateralization of language function for presurgical evaluation.^7^, ^8^

Pharmaco‐fMRI is a promising emerging application to probe pharmacological effects on functional neuroanatomy, and can help to determine early treatment response, mechanisms of drug efficacy, and side effects.^9^, ^10^, ^11^ Our recent studies showed that both valproate (VPA) and levetiracetam (LEV) may lead to a normalization of the altered fMRI activation patterns in genetic generalized^1^, ^12^and focal epilepsies,^1^, ^2^, ^13^ respectively. Previous cognitive fMRI studies probing the effects of topiramate (TPM),^1^, ^14^, ^15^, ^16^, ^17^, ^18^, ^19^ which is often associated with cognitive impairment and improvement of psychometric measures after dose reduction or discontinuation,^3^, ^4^, ^20^, ^21^, ^22^ and zonisamide (ZNS), which has similar but less pronounced cognitive side effects,^5^, ^6^, ^7^, ^22^, ^23^ highlighted the following patterns of dysfunctional fMRI activations: (1) decreased activation in task‐positive regions, that is, dominant inferior and middle frontal gyri (IFG and MFG); and (2) failure to deactivate task‐negative regions, including the DMN.^7^, ^8^, ^19^ TPM was particularly related to impaired attenuation of language‐associated deactivation.^9^, ^10^, ^11^, ^19^

Relatively few studies^24^, ^25^ have investigated the fMRI correlates of CBZ and LTG in patients with epilepsy. The aim of this retrospective study was to assess how LTG and CBZ influence task‐related fMRI activation or deactivation patterns, and to improve our understanding of medication‐specific effects on cognitive functional anatomy. To control for the effects of comedication and disease‐related factors, we used LEV‐treated patients as a “patient control” group, owing to its normalizing effects on cognitive fMRI paradigms; patients on LEV showed similar task‐related deactivation patterns to healthy controls.^13^ We used healthy control subjects (CTR) to detect common cognitive effects of different AEDs. We hypothesized that impaired verbal fluency performance is associated with failure either to activate task‐positive or to deactivate task‐negative regions as assessed by a language fMRI paradigm.

## 2 MATERIALS AND METHODS

### 2.1 Participants

From our clinical verbal fluency fMRI database, we selected patients with drug‐refractory epilepsy who had undergone clinical language fMRI scans at the Chalfont Centre for Epilepsy (UK) between March 2010 and March 2017 as part of their presurgical evaluation. All patients were adults and had been seen at the adult epilepsy clinics of the National Hospital for Neurology and Neurosurgery and Chalfont Centre for Epilepsy. We included patients who were taking 1 of the following 3 AEDs: CBZ, LTG, or LEV, either alone or in combination with other AEDs, but not with other sodium channel blockers (lacosamide and/or oxcarbazepine), TPM, or ZNS.^19^ We also excluded patients who were receiving treatment with psychotropic medications, such as antidepressant and antipsychotic agents.

All patients had to be fluent in English and able to understand the task instructions for testability with the language fMRI task. We could not control for task compliance, because our standard clinical verbal fMRI paradigm is conducted covertly. Hence, we excluded all patients without activations of language‐relevant regions (IFG and MFG) from the analysis. Also, we excluded patients with data acquired postoperatively and those with large lesions or tumors (>2 cm) to avoid problems with imaging normalization and further statistical analysis.

Seventy‐four patients on LEV, 68 on LTG, and 51 on CBZ were eligible. Starting with the LEV and LTG groups, we propensity‐matched (PSM) CBZ patients for the variables of age at scan, age at onset, sex, language laterality index (LI), total number of medications, localization, and localization laterality, using SPSS version 22.0 (IBM, Armonk, NY, USA). We included 42 patients in each AED group in the final analysis. We further matched 42 English‐speaking CTR, recruited from the local community, using the variables age, gender, and language laterality index in the PSM analysis for 3 AED groups.

### 2.2 MRI data acquisition and fMRI paradigm

Gradient echo‐planar images providing blood oxygen level–dependent (BOLD) contrast were acquired on a 3‐T Excite HDx scanner (General Electric, Milwaukee, WI, USA), using a standard 8‐channel receive coil. Each volume comprised 50 contiguous oblique axial slices, ensuring full brain coverage, with 2.5‐mm slice thickness, 64 × 64 matrix, and 24‐cm field of view, providing an in‐plane voxel size of 3.75 × 3.75 mm. Echo time was 25 milliseconds, and repetition time was 2.5 seconds. A scanner upgrade had taken place in 2013, but the scanning protocol remained the same. Patients performed a covert verbal fluency task lasting for 5 minutes. During the paradigm, 30‐second blocks of task were alternated with 30‐second blocks of crosshair fixation as a control condition. Patients were instructed to covertly generate words starting with a visually presented letter (A, D, E, S, W).

### 2.3 MRI data analysis

fMRI data were preprocessed with Statistical Parametric Mapping 8 (SPM8) toolkit, version 6313 (http://www.fil.ion.ucl.ac.uk/spm/), which included realignment, spatial normalization to the Montreal Neurological Institute (MNI) template supplied by SPM8, resampling (isotropic 3 × 3 × 3 voxels), and spatially smoothing (8 mm). We performed the statistical fMRI analyses at the first level (single subject), and then at the group level. At the subject level, the task was modeled by convolving the vector of block onsets with a canonical hemodynamic response function to create regressors of interest, and motion parameters were included as confounds. Contrast images for each participant were created for task‐relevant activation and deactivation. For the second‐level analysis, we introduced CTR and entered activation contrasts for each patient and control into a full factorial design with group as a factor (CBZ, LEV, LTG, CTR) and age, gender, and LI as regressors. All disease‐related variables used in the PSM analysis were entered as regressors of no interest for patient‐to‐patient group comparison (CBZ, LEV, and LTG). An exploratory statistical threshold was set at *P* < .005 uncorrected with a 10‐voxel minimum cluster size extent threshold.^1^, ^26^ To be able to examine whether group differences were related to activation or deactivation, we masked the results with a binarized average task activation map of the controls and then with the binarized deactivation map to include the contrast‐relevant brain areas.

Interpretation of the results at the subject level and group level was not blinded, because resultant maps represent *t* maps at a predefined statistical threshold. We anatomically objectified peak activations from group comparisons with coordinates in the MNI template.

### 2.4 Language Lateralization Index

Laterality indices (LIs) of statistic parametric maps were measured to quantitatively assess hemispheric dominance for language.^27^ For each subject, we used the bootstrap method of the lateralization index toolbox in SPM8^28^ on verbal fluency statistic parametric maps. In accord with previous studies,^29^, ^30^ activated voxels in IFG and MFG for the verbal fluency paradigm were computed according to the formula LI = (L − R)/(L + R), where L = left and R = right. A positive LI indicates left hemispheric dominance and a negative index indicates right hemispheric lateralization. LIs were subsequently classified as left‐hemisphere dominant (LI > +0.2); atypically dominant, comprising a bilateral distribution (−0.2 ≤ LI ≤ +0.2); and right‐hemisphere dominant (LI < −0.2).^29^, ^31^ We matched the groups for language LIs to control for differences in hemispheric language dominance.

### 2.5 Neuropsychological measures, correlation with language networks, and dose dependence of the patterns of activation and deactivation

Out‐of‐scanner neuropsychological results are available in some participants (Table 1). This mainly included tests measuring language function: verbal and category fluency tests, National Adult Reading Test to provide an estimate of general intellectual level (intelligence quotient [IQ]), and McKenna Graded Naming Test of expressive language functions. In these participants, post hoc analyses were conducted correlating these scores with the task‐activation patterns. Dose dependence was conducted correlating the dose of each patient of different AED group with task‐activation/deactivation patterns. Positive and negative correlations were explored using these parameters as regressors in 1‐sample *t* tests over the whole brain for each language task.^13^, ^32^ The level for significance was *P* < .005 uncorrected with a 20‐voxel threshold extent.

**Table 1:** Demographic and neuropsychological measures

Demographic and clinical details	On LEV, n = 42	On LTG, n = 42	On CBZ, n = 42	CTR, n = 42	*P*
Age, y, median (range)	36.5 (18‐66)	34.5 (16‐72)	37.5 (23‐69)	35.5 (21‐64)	.713
Gender, F/M	25/17	21/21	19/23	21/21	.613
Handedness, R/L/A	33/8/1	37/5/0	37/5/0	38/4/0	.596
Laterality index, median (range)	0.76 (−0.66 to 0.98)	0.74 (−0.83 to 1)	0.62 (−0.85 to 0.99)	0.80 (−0.66 to 1)	.394
Age at onset, y, median (range)	16.8 (1‐49)	15.0 (1‐49)	14.3 (1‐40)		.319
Duration, y, median (range)	19.0 (3‐51)	18.0 (4‐28)	20.5 (4‐62)		.207
Pathology, temporal/frontal/parietal/unknown	32/8/1/1	32/10/0/0	31/10/1/0		.735
Pathology lateralization, L/R/bilateral/unknown	22/13/6/1	21/12/6/3	15/18/9/0		.296
Scanner, original/upgraded	26/16	22/20	25/17		.655
AEDs, median (range)	2 (1‐4)	2 (1‐4)	2 (1‐3)		.411
Mono‐/polytherapy, n	13/29	13/29	13/29		1
Monotherapy	13	13	13		.609
2 AEDs	16	16	22		
3 AEDs	12	8	7		
4 AEDs	1	2	0		
Dose, mg, median (range)	2000 (375‐4000)	300 (50‐800)	1000 (200‐1600)		

Pearson χ^2^ was used for dichotomous variables, and Kruskal‐Wallis test was used for all other variables (*P* < .05).

A, ambidextrous; AED, antiepileptic drug; CBZ, carbamazepine; CTR, healthy control subjects; F, female; L, left; LEV, levetiracetam; LTG, lamotrigine; M, male; R, right.

### 2.6 Statistical analysis of clinical, demographic, and cognitive measures

Statistical analysis of clinical and behavioral data was conducted with SPSS 22.0. For normally distributed data, we used 1‐way analysis of variance and 2‐sample *t* test for post hoc analysis with a significance threshold of 0.05 (Bonferroni‐corrected). We applied χ^2^ tests to categorical data, and the Kruskal‐Wallis or Mann‐Whitney *U* test to all other measures with a statistical significance threshold of .05 (Bonferroni‐corrected).

### 2.7 Standard protocol approvals, registrations, and patient consent

This study was approved by the Joint Ethics Committee of the National Hospital for Neurology and Neurosurgery and University College London Institute of Neurology. The Research Ethics Committee classified this work as evaluation of clinical services (routine language fMRI), and therefore individual consent from patients was not needed.

## 3 RESULTS

Table 1 provides further demographic and clinical details of subjects. Appendix S1 presents the details of AEDs for each patient in this study.

### 3.1 Cognitive performance

There were significant group differences in cognitive test performance for verbal and category fluency tests (Table 2). Post hoc group comparisons revealed that all 3 AED groups performed worse in letter fluency than CTR (CBZ, *P* < .001; LEV, *P* = .002; LTG, *P* = .029). For category fluency, this was demonstrated for the CBZ group (*P* < .001). There was no statistical difference between LEV‐ and LTG‐treated patients, either in letter (*P* = .299) or category (*P* = .525) fluency, and both LEV and LTG groups performed better than patients on CBZ for both fluency measures (letter fluency: LEV vs CBZ, *P* = .015; LTG vs CBZ, *P* = .004; category fluency: LEV vs CBZ, *P* = .05; LTG vs CBZ, *P* = .012).

**Table 2:** Cognitive measures

Measure	LEV	LTG	CBZ	CTR	*P*
Letter fluency, mean (SD)	n = 34, 12.85 (4.6)^a^	n = 37, 14.16 (5.8)^a^	n = 36, 10.28 (5.2)	n = 36 17.75 (7.8)	.000
Category fluency, mean (SD)	n = 36, 17.03 (5.8)^a^	n = 37, 17.92 (6.1)^a^	n = 36, 14.31 (5.8)	n = 36, 19.42 (9.0)	.016
McKenna Graded Naming Test, median (range)	n = 25, 16 (7‐27)	n = 29, 15.5 (6‐26)	n = 34, 14 (3‐25)	–	.346
National Adult Reading Test, median (range)	n = 25, 98 (36‐119)	n = 29, 100 (70‐125)	n = 24, 97.5 (27‐117)	–	.244

For letter and category fluency, analysis of variance was used to detect differences between groups at *P* < .05.CBZ, carbamazepine; CTR, healthy control subjects; LEV, levetiracetam; LTG, lamotrigine; SD, standard deviation.

a Post hoc group comparisons (2‐tailed *t* test, *P* < .05) revealed that both LEV and LTG groups performed better on letter and category fluency than the CBZ group, and all 3 patient groups performed worse than healthy controls. For McKenna Graded Naming Test and National Adult Reading Test, healthy control data were not available. Nonparametric testing (Kruskal‐Wallis test) did not show any differences between the 3 different patient groups.

### 3.2 Language Lateralization Index

Five LTG‐treated, 5 CBZ‐treated, and 2 LEV‐treated patients as well as 4 CTR exhibited right‐hemisphere language dominance, whereas 1 LTG‐treated, 2 CBZ‐treated, and 3 LEV‐treated patients had bilateral language representations. Mann‐Whitney *U* test for independent samples did not indicate a significant difference in distribution of LIs across the 3 groups (*U* = 877.5, *P* = .96). There was no correlation of lateralization indices with age at onset, disease duration, or number of comedications (*P* > .05).

### 3.3 fMRI results

**Figure 1:**
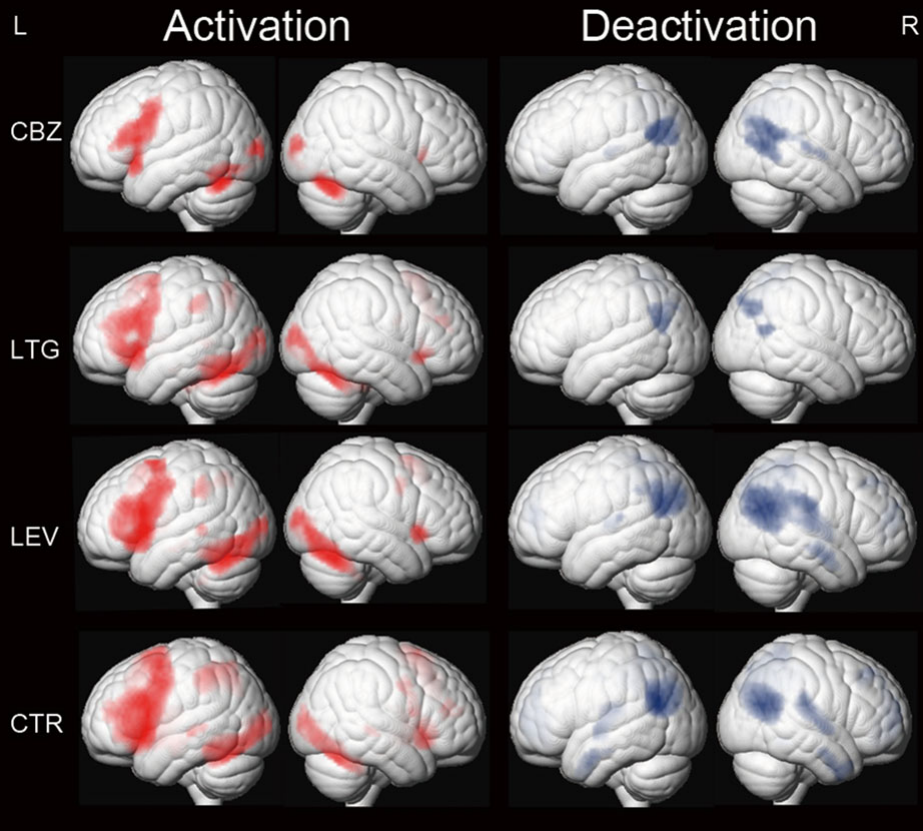
One‐sample *t* tests of functional magnetic resonance imaging activation and deactivation maps for the 3 different patient groups on carbamazepine (CBZ), levetiracetam (LEV), and lamotrigine (LTG) and healthy controls (CTR) are demonstrated on a surface‐rendered brain template. Task‐relevant regions (red) include bilateral inferior and middle frontal gyrus (left > right), bilateral supplementary motor area, the left dorsolateral parietal region, and bilateral inferior occipital lobes. Areas of task‐related deactivations (blue) include the bilateral precuneus, posterior cingulate, angular gyrus, and medial prefrontal and lateral temporal cortex. *P* < .005, 20‐voxel threshold extent. L, left; R, right

One‐sample *t* tests of task‐relevant activations and deactivations of each group are demonstrated in Figure 1. Areas of significant activations were observed in frontal language areas, including the IFG, MFG, bilateral supplementary motor areas (SMAs), and left lateral parietal region. Deactivated areas of the DMN included bilateral precuneus, posterior cingulate, angular gyrus, and medial prefrontal and lateral temporal cortices.

### 3.4 Comparison of TPM, ZNS, and LEV groups

**Figure 2:**
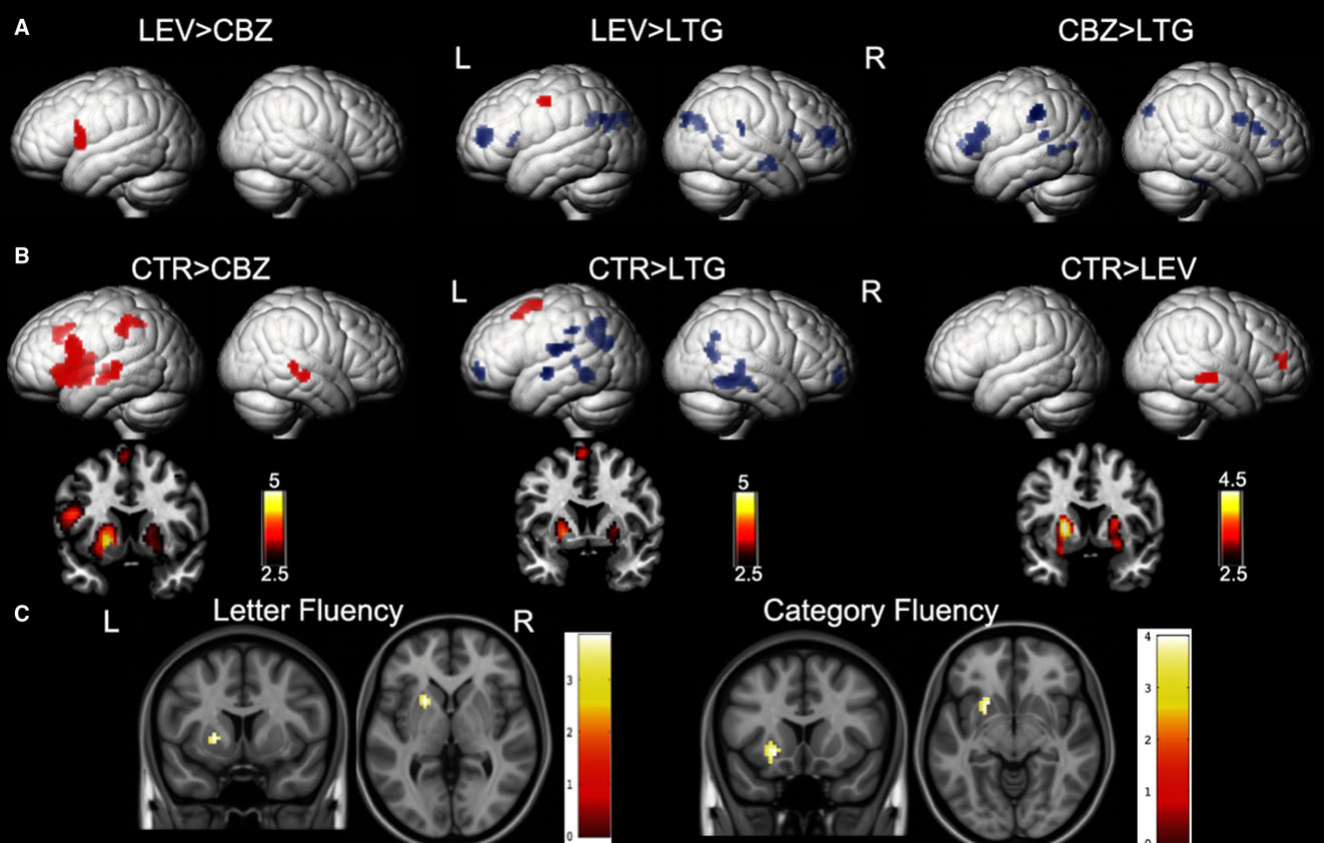
Significant group differences between patients on carbamazepine (CBZ), levetiracetam (LEV), and lamotrigine (LTG) and healthy controls (CTR) are demonstrated on a surface‐rendered brain template, and the subcortical changes are demonstrated on a coronal brain template with a bar chart indicating *z* score. A, Patients on CBZ activated less in the left inferior frontal gyrus than patients on LEV. In patients on LTG, deactivation was reduced in the task‐negative areas, including the middle frontal gyri and left dorsal parietal region of the precuneus, compared with patients on LEV and CBZ. *P* < .005, 10‐voxel threshold. B, All 3 groups of patients activated the putamen less bilaterally (left > right) than CTR. Patients on CBZ activated the left inferior frontal gyrus less than CTR. There was less deactivation in the bilateral medial frontal gyri and bilateral lateral temporal regions, left rolandic operculum, and left inferior parietal lobule in patients on LTG. *P* < .005, 10‐voxel threshold. C, The clinical neuropsychological performance on letter fluency (143) and category fluency (145) in 4 groups together positively correlates with activation in the left putamen. *P* < .005, 20‐voxel threshold. L, left; R, right

Compared to patients taking LEV, CBZ‐treated patients showed less activation of left IFG (Figure 2A; Table 3). LTG‐treated patients exhibited fewer task‐related deactivations in bilateral frontal and parietal regions, as well as in the right middle temporal gyrus (Figure 2A; Table 3).

**Table 3:** Anatomic description and peak activations of resultant areas from group comparisons

Region	MNI coordinates, x, y, z	*Z* score	*P*
Patients on LEV > patients on CBZ			
Left inferior frontal gyrus	−60, 11, 7	3.37	<.001
Patients on LEV < patients on LTG			
Left cuneus	−6, −76, 25	3.43	<.001
Left medial frontal gyrus	−6, 50, 13	3.39	<.001
Left precuneus	−9, −55, 31	3.14	.001
Left angular gyrus	−45, −67, 31	2.81	.003
Right hippocampus	27, −13, −17	2.89	.002
Right middle temporal lobe	48, −4, −20	2.87	.002
Right calcarine	15, −58, 7	2.68	.004
Patients on LEV > patients on LTG			
Left precentral gyrus	−57, −13, 49	3.35	<.001
Patients on CBZ < patients on LTG			
Left inferior parietal lobe	−39, −37, 37	3.26	.001
Left cuneus	0, −88, 48	3.17	.001
Left calcarine	−12, −73, 7	2.8	.003
Right anterior cingulate cortex	9, 20, 22	2.85	.002
Healthy controls > patients on CBZ			
Left putamen	−18, 11, −2	4.5	<.001^a^
Left inferior frontal gyrus	−51, 14, 19	4.14	<.001
Left supplementary motor area	−3, 20, 46	3.53	<.001
Left middle temporal lobe	−63, −25, −5	3.14	.001
Left thalamus	−9, −16, 4	3.28	.001
Left inferior parietal lobe	−51, −37, 46	2.95	.002
Right putamen	21, 20, 2	3.65	<.001
Right middle temporal lobe	60, −25, −8	3.2	.001
Healthy controls > patients on LEV			
Left putamen	−21, 8, 4	4.13	<.001
Right putamen	24, −10, −14	3.73	<.001
Right middle temporal lobe	60, −22, −8	3.36	<.001
Right middle frontal gyrus	30, 50, −40	2.9	.002
Healthy controls > patients on LTG			
Left putamen	−21, 8, −2	3.98	<.001
Left supplementary motor area	−3, 2, 67	3.37	<.001
Right putamen	24, 5, 1	3.06	.001
Healthy controls < patients on LTG			
Left precuneus	−15, −55, 40	3.68	<.001
Left rolandic operculum	−39, −19, 22	3.41	<.001
Left fusiform	−30, −49, −8	3.18	.001
Right medial frontal gyrus	3, 62, −8	3.45	<.001
Right calcarine	12, −61, 13	3	.001

Coordinates are given in MNI space.

CBZ, carbamazepine; LEV, levetiracetam; LTG, lamotrigine; MNI, Montreal Neurological Institute.

a Left putamen activations are shown corrected for multiple corrections in healthy controls versus patients on CBZ, familywise error, *P* < .05.

### 3.5 Comparison of patient groups with CTR

Compared with CTR, all 3 AED groups showed less activation of the bilateral putamen. Available out‐of‐scanner verbal and category fluency scores positively correlated with activation in left putamen in all participants (patients and controls): the higher the scores, the higher the activation in left putamen (Figure 2C). In addition, CBZ‐treated patients showed reduced activation in left IFG, left middle temporal lobe, left inferior parietal lobe, left SMA, and right middle temporal lobe, which are mainly in task‐positive network regions (Figure 2, Table 3); LTG‐treated patients showed impaired deactivation in temporal and frontal areas bilaterally and in left parietal lobes, which are mainly task‐negative networks, and less activation in left SMA (Figure 2, Table 3). All control scans were performed prior to the scanner upgrade in 2013. Patients scanned prior to the upgrade were matched to controls for each AED group (CBZ, n = 25; LTG, n = 22; LEV, n = 25), and the group differences were similar to those above. In each AED group, the patterns of activation and deactivation did not correlate with dosage accordingly (*P* > .05).

## 4 DISCUSSION

We investigated language paradigm‐specific fMRI effects of 2 commonly used AEDs in focal epilepsy; CBZ‐treated patients activate less the left IFG, whereas LTG‐treated patients fail to deactivate task‐related areas compared with either patients receiving LEV or CTR.

Patients on CBZ showed dysfunctional network activation in the left IFG, which was not observed in patients on LTG, and which relates to poorer out‐of‐scanner performance on fluency tasks in accordance with previous cognitive findings.^1^, ^2^ The first pharmaco‐fMRI study in temporal lobe epilepsy^24^ reported reduced fMRI activation within the medial temporal lobe during a memory task, which correlated with CBZ serum levels: the higher the CBZ levels, the lower the fMRI activation. In a recent study on brain connectivity,^25^ CBZ treatment had a region‐specific effect on the limbic circuit and thalamus network. In our previous pharmaco‐fMRI studies,^19^ we observed impaired task‐related fMRI activation, including Broca's region as well as dorsolateral frontal and parietal cortices activation, and poorer performance on verbal fluency in patients on TPM and ZNS. Dorsolateral frontal and parietal cortices, activated by the verbal fluency task, are also part of extended functional networks implicated in working memory and attention.^33^ TPM and ZNS have frequently been linked with higher‐level cognitive dysfunction, whereas CBZ seems to cause relatively less apparent cognitive impairments. In this study, unlike TPM and ZNS, decreased activation in CBZ‐treated patients was confined to the left IFG.

Similar to patients on TPM,^19^ we show that LTG‐treated patients failed to deactivate parts of the frontal and parietal cortices including medial frontal gyrus and cuneus and precuneus, which are nodes of the DMN.^4^ This matches with previous results demonstrating that successful task execution has been associated with effective deactivation of task‐negative areas.^4^, ^34^ In our recent study,^19^ TPM‐treated patients failed to deactivate task‐relevant DMN nodes compared to patients taking ZNS or LEV, which was associated with more cognitive impairment than those on ZNS. Moreover, direct comparison to ZNS shows that TPM led to a failure to deactivate DMN nodes of language task on the right hemisphere. ZNS treatment leads to similar but less pronounced impairment than TPM in epilepsy patients.^22^, ^23^ Our LTG‐treated patients had similar out‐of‐scanner verbal fluency performance to LEV‐treated patients, and both LTG‐ and LEV‐treated patients performed better than patients on CBZ. We suggest that the failure to deactivate task‐relevant DMN nodes might also affect language performance, but to a lesser degree than reduced activation in Broca's area.

Interestingly, we found an effect of all AEDs on activations in the putamen bilaterally, when compared with controls. The greater the activation of the left putamen, the better were out‐of‐scanner letter and category fluency scores. These findings in patients are in line with a suspected role of the left putamen in speech production and processing,^35^ and may account for the negative effect all AEDs have on cognitive processing. At the same time, subcortical structures, like the putamen, also play important roles in ictogenesis,^36^ which might reflect a common site for the antiseizure effects of AEDs. With the present study design, it is difficult to ascribe these effects to the effect of AEDs or the influence of seizure activity. Future prospective longitudinal studies with well‐controlled seizure characteristics in AED monotherapy will help further address the underlying mechanisms.

We included LEV‐treated patients as a “patient control group.” Our recent fMRI studies^13^, ^19^ showed a “normalizing” effect of LEV on cognitive networks without necessarily improving cognition. Task‐ and syndrome‐specific regional fMRI effects as well as dose dependency were demonstrated with a verbal and visual‐spatial working memory task. Patients on LEV displayed an augmentation of task‐related deactivation in the affected temporal lobe compared to patients without LEV. In the present study, we also did not detect abnormal deactivation patterns in LEV patients, replicating previous findings of normalization of deactivation patterns on LEV,^13^, ^19^ which justifies our inclusion of LEV‐treated patients as a patient control group. We argue that “normalization, or more pronounced deactivation patterns” may represent a beneficial drug effect, as data in healthy subjects and epilepsy patients show that progressive deactivation of the DMN nodes during cognitive tasks is associated with improved performance.^37^

The strengths of this study include the large sample size, well‐matched disease characteristics, and introduction of healthy controls as a comparison group. Our study is still limited by its retrospective nature. The statistical threshold (*P* < .005 with 10‐voxel threshold extent) used for the second‐level analysis was uncorrected but enables an exploratory view of the differences between AED treatment groups, with peak activations within implicated regions almost all reaching *P* < .001 uncorrected (Table 3). We precisely matched participants to reduce factors that would impact results, but still the majority of patients were receiving multiple AEDs (mainly 2 compounds; 2 were on 4 AEDs). This may have contributed to poor cognitive performance and may represent an additional source of variance. In addition, AED plasma concentrations were not examined at the time of the scan. Recent research has shown that every additional AED leads to further cognitive impairment.^38^ We tried to control for the effect of comedication as much as possible, by excluding medications that are known to affect the activation patterns, such as TPM and ZNS^19^ and other sodium‐channel blockers (oxcarbazepine and lacosamide). In addition, we propensity‐matched groups for the number of AEDs, and as an additional measure, individual AED comedication was included as a regressor of no interest in the fMRI analysis model, a standard methodology in fMRI analysis. Out‐of‐scanner cognitive data were not available in all patients. All patients had tried multiple AED treatments but still suffered from refractory epilepsy. The effect of seizures on our findings, however, could not be quantified in terms of frequency or severity.

Identifying language lateralization with fMRI is crucial for risk assessment during presurgical evaluation.^8^ It would thus be important to establish whether fMRI changes due to AEDs can lead to altered lateralization of language. As groups were matched for LIs, we cannot comment on the potential effects of CBZ and LTG on language lateralization. Although the number of AEDs taken by an individual was not correlated with LIs, the specific effects of single AEDs would be more appropriately investigated by longitudinal studies carried out before and after treatment initiation.

Together with our previous findings,^13^, ^19^ patients on CBZ and ZNS showed similar dysfunction within task‐related activation networks, which was associated with poorer performance in verbal fluency. Patients on LTG only presented with abnormal deactivation, associated with limited impact on language function. Patients on TPM showed both decreased task‐relevant frontal activation and abnormal deactivation of task‐negative networks, which was associated with the most pronounced impairment of language and working memory.^19^, ^23^ In contrast, both VPA and LEV appear to restore the fMRI activation patterns in genetic generalized^12^ and focal epilepsies,^13^ respectively. These fMRI verbal fluency activation and deactivation patterns are not dose‐dependent. In our previous study,^13^ dose dependence was specific to memory in LEV patients and was more apparent in a more demanding nonverbal task than in a verbal memory task. Hence, it suggests that dose dependence may not be obvious in a relatively “pure” verbal fMRI task. Although our observed results are only robust with high sensitivity in group comparisons, they could provide valuable information for interpreting clinical language fMRI scans in a variety of patients. There were no differences in the National Adult Reading Test, which is considered to reflect comorbid IQ. Thus, it is unlikely that our results are due to an uncontrolled sample bias with patients with lower cognitive abilities choosing the older AED. The observed BOLD changes provide testable hypotheses for intrasubject initiation and withdrawal studies of AEDs, as shown previously for TPM.^16^ Prospective, longitudinal studies with cognitive fMRI and neuropsychological data collection before and after the initiation or withdrawal of these AEDs may be instrumental to better characterize AED‐specific effects and disentangle the effects of different AEDs on epileptic and functional language networks.

## AUTHOR CONTRIBUTIONS

F.X.: drafting/revising the manuscript, analysis and interpretation of data, and statistical analysis. L.C.: drafting/revising the manuscript, analysis and interpretation of data, and statistical analysis. B.W.: drafting/revising the manuscript, interpretation of data, and statistical analysis. M.S.: drafting/revising the manuscript, data collection. G.W.: drafting/revising the manuscript, data collection. J.B.: drafting/revising the manuscript and data collection. K.T.: drafting/revising the manuscript and data collection. A.H.: drafting/revising the manuscript and data collection. C.V.: drafting/revising the manuscript and data collection. S.B.V.: drafting/revising the manuscript and interpretation of data. S.O.: drafting/revising the manuscript and interpretation of data. D.Z.: drafting/ revising the manuscript and study supervision. J.W.S.: drafting/revising the manuscript, study concept or design, interpretation of data, and study supervision. P.J.T.: drafting/revising the manuscript, study concept or design, interpretation of data, and study supervision. J.S.D.: drafting/ revising the manuscript, study concept, interpretation of data, and study supervision. M.J.K.: drafting/revising the manuscript, study concept or design, interpretation of data, and study supervision.

## DISCLOSURE OF CONFLICT OF INTEREST

F.X., L.C., B.W., J.B., M.S., K.T., G.W., C.V., S.B.V., A.H., S.O., D.Z., and P.J.T. do not have any conflict of interest to disclose. J.W.S. has received honoraria and grants from UCB, Eisai, Teva, Lundbeck, and GSK. J.S.D. serves on the scientific advisory boards for and/or has received funding for travel from GE Healthcare, GSK, Eisai, and UCB. M.J.K. served on a scientific advisory board of GE Healthcare and has received honoraria for lectures from Eisai, Bial, Novartis, and UCB. We confirm that we have read the Journal's position on issues involved in ethical publication and affirm that this report is consistent with those guidelines.
